# Detection of clinically and mammographically occult cancer by bilateral whole breast ultrasound: experience from a district general hospital

**DOI:** 10.1186/bcr2981

**Published:** 2011-11-04

**Authors:** TR Rehman, B Upadhyay, L Kanagarajah

**Affiliations:** 1 Basildon and Thurrock University Hospital, Basildon, UK

## Introduction

Although ultrasound is considered to be a poor screening tool, it is reported to be useful in detecting multifocal cancers [1]. This study aims to look at the use of bilateral whole breast ultrasound in detecting clinically and mammographically occult breast cancers.

## Methods

All patients presenting to symptomatic clinic in Basildon Hospital underwent sonographer-performed bilateral whole breast ultrasound before the year 2010. All breast cancer patients in 2009 whose imaging details are available on the Radiology Management System (RMS) and PACS are the materials of this study (number of patients: 101). Clinical and imaging details were evaluated on RMS and PACS and cases in which cancer was detected only by ultrasound were identified.

## Results

Among the 101 patients, multifocal cancers were detected in ipsilateral breast in two patients. Both of these patients had one clinically and mammographically detected cancer. The second smaller lesion was depicted only on ultrasound. Apart from the above multifocal cancers, there was no case in which cancer was identified only on ultrasound.

## Conclusion

Operator-performed bilateral whole breast ultrasound is useful in detecting multifocal primary breast cancer although its routine use in all the patients is yet to be proven.
